# Selection and Functionalization of Germanium Nanowires for Bio-Sensing

**DOI:** 10.1021/acsomega.2c04775

**Published:** 2022-09-23

**Authors:** Siriny Laumier, Thomas Farrow, Harm van Zalinge, Luca Seravalli, Matteo Bosi, Ian Sandall

**Affiliations:** † Department of Electrical Engineering and Electronics, University of Liverpool, 9 Brownlow Hill, Liverpool L69 3GJ, U.K.; ‡ Institute of Material for Electronic and Magnetism, Parco Area delle Scienze 37/A, 43124 Parma, Italia

## Abstract

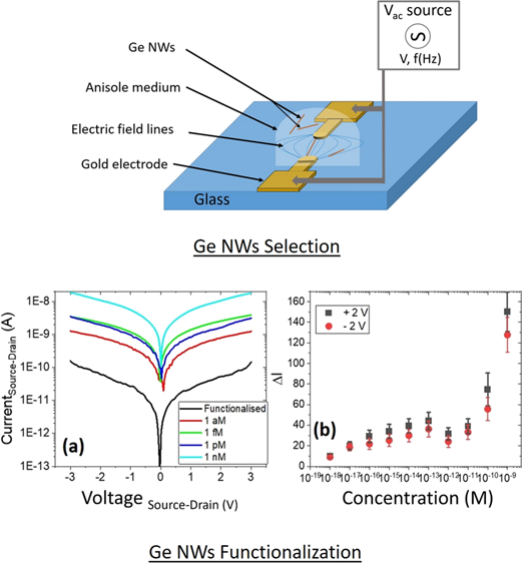

In this paper, we investigate the use of dielectrophoresis to align germanium nanowire arrays to realize nanowire-based diodes and their subsequent use for bio-sensing. After establishing that dielectrophoresis is a controllable and repeatable fabrication method to create devices from germanium nanowires, we use the optimum process conditions to form a series of diodes. These are subsequently functionalized with an aptamer, which is able to bind specifically to the spike protein of SARS-Cov2 and investigated as a potential sensor. We observe a linear increase in the source to drain current as the concentration of spike protein is increased from 100 fM/L to 1 nM/L.

## Introduction

Recently one-dimensional semiconductor systems, such as nanowires (NWs), have been widely investigated for chemical, optical, and biological sensors.^1−4^ The use of NWs provides dimensions comparable to those of biomolecules and cellular structures, while the large surface-to-volume ratio enables the NWs to be highly sensitive and responsive to biochemical changes in the environment.^5^

Despite their promising potential as a platform for bio-sensing, development and exploitation of NWs has been limited due to issues in fabricating reliable and repeatable devices. The main techniques currently used to realize NW-based devices and biosensors are nonideal. Typical top-down approaches use high-resolution lithography and plasma etch-based tools to define and pattern horizontal NWs on a substrate.^6,7^ On the other hand, bottom-up approaches are much cheaper and easier, typically involving drop-casting of NWs between two predefined electrodes.^8,9^ However, given the nonuniformity of the NWs and the random orientation of their deposition between the electrodes, many NWs will not be contacted; there are also issues associated with devices containing multiple NW-to-NW junctions. These problems make it difficult to model and understand the final operation and performance of a device. Additionally, in both fabrication approaches, it is difficult to obtain devices with repeatable performance due to the intrinsic differences (i.e., conductivity, defects, doping profile, etc.) between NWs grown in an ensemble or due to slight differences in the patterning and etching of the NWs.

An alternative approach, which can solve some of these issues, is the use of dielectrophoresis (DEP): it enables self-alignment of NWs across a predefined electrode gap and also offers the possibility to select ensembles of NWs with specific properties for a given application. Moreover, it is low-cost and permits a rapid fabrication of devices.^10^ DEP uses a force applied by a nonuniform electric field on a conductive or dielectric particle. This force can be exploited to manipulate a range of molecules and particles.^11−14^

**Figure 1:**
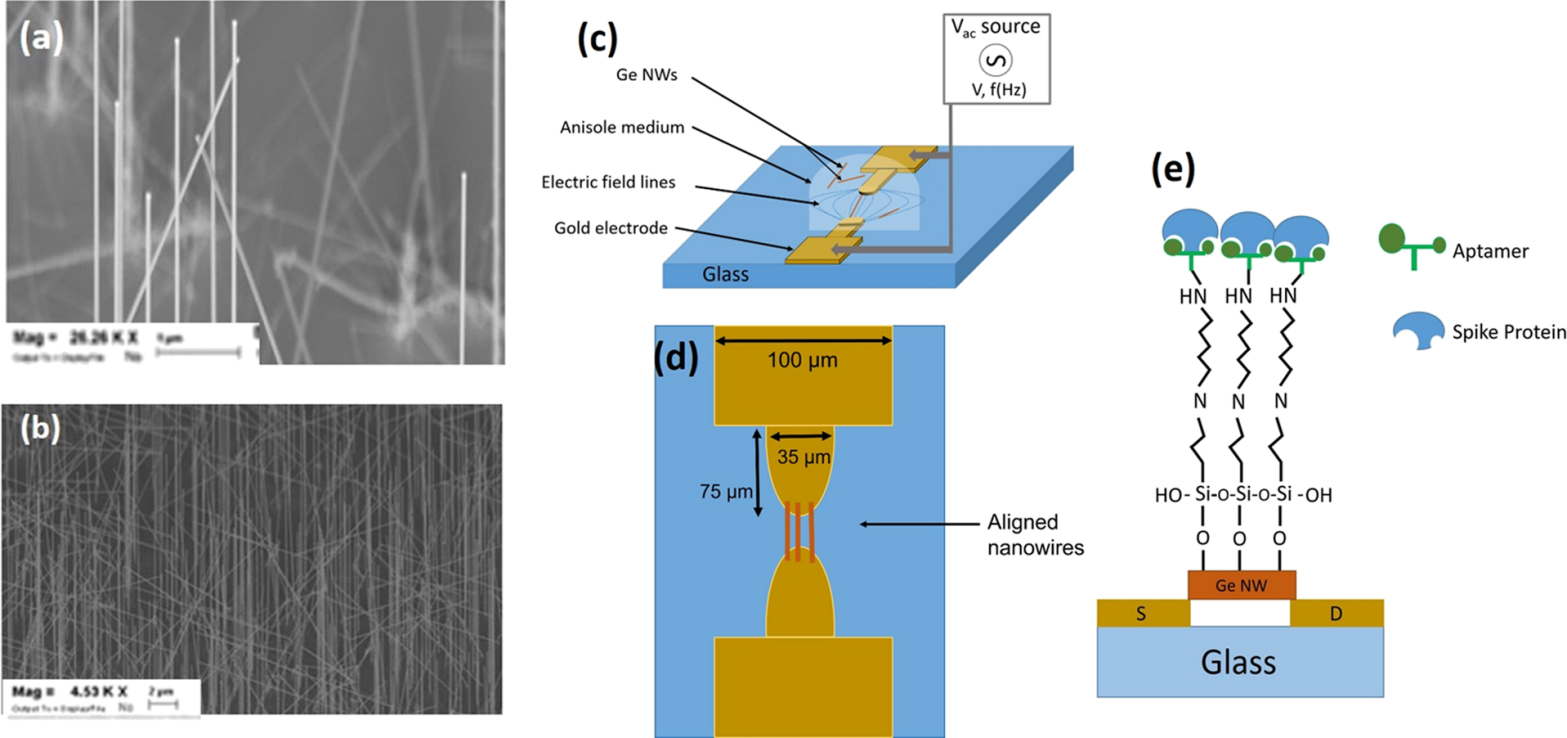
(a) and (b) SEM images of typical as-grown Ge NWs, (c) overview schematic of DEP system (not to scale), (d) top view schematic of final device (not to scale), and (e) side profile of the final device with functionalization (not to scale).

Ge NWs were grown on Si substrates by metal–organic vapor-phase epitaxy (MOVPE) using Au nanoparticles as seeds: representative scanning electron microscopy (SEM) images are shown in Figure 1a,b. Further details on the growth and properties of these nanostructures can be found in the Methods section. Previous work on silicon NWs^10^ has shown that this approach can lead to the realization of NW-based field-effect transistors. Furthermore, it has been shown that as the frequency of the applied AC field is increased, and only highly conductive NWs are aligned: for this reason, the technique results in superior device performance, because lower quality NWs are removed from the ensemble.

The basic design of an immuno-FET is a transistor in which the gate is replaced by a layer of antibodies specific to the target protein. Once the target is attached to the antibody, the charged areas of the protein will cause a change in the conduction channel of the FET and hence a change in the conductivity between the source and drain. NWs have been used previously by a number of groups^15−17^ as immuno-FETs (although traditionally fabricated via other techniques).

Currently, a major restriction for the use of immuno-FETs is the screening effect when employed in physiological fluids. The ions present in the solution cause the formation of a double layer with a thickness equal to the Debye length. Any change in the charge distribution outside this layer will not affect the conduction channel of the FET. In recent years, a new group of molecules has been developed. These so-called aptamers consist of short DNA, RNA, or peptide strands.^18,19^ Utilizing their conformation and charge distribution, the aptamers are capable of specifically binding to individual proteins. As they are significantly smaller than antibodies, the result is that a protein bound via an aptamer to the conduction channel will create a change in the electrical properties at higher salt concentration. In addition, aptamers are significantly cheaper and more stable than antibodies.

While FETs have previously been demonstrated from NWs, in this work, we have made three distinct changes. First, we are utilizing germanium (Ge). The higher conductivity of Ge (high carrier mobility, and small band gap) is anticipated to result in an enhanced sensitivity to protein binding events, as previously noted for Ge quantum dots.^20−22^ Other advantages of germanium in comparison with silicon include higher carrier injection velocity and lower temperature growth and processing, making its choice attractive for the development of CMOS-compatible devices.^23^ The second difference is in utilizing DEP as a fabrication method for the FETs. The third difference is in the use of aptamers rather than antibodies to attach the target protein. While we are using SARS-Cov2 here as an example of a rapidly emerging threat, we envisage that the principle can be extended to any protein.

## Results and Discussion

**Figure 2:**
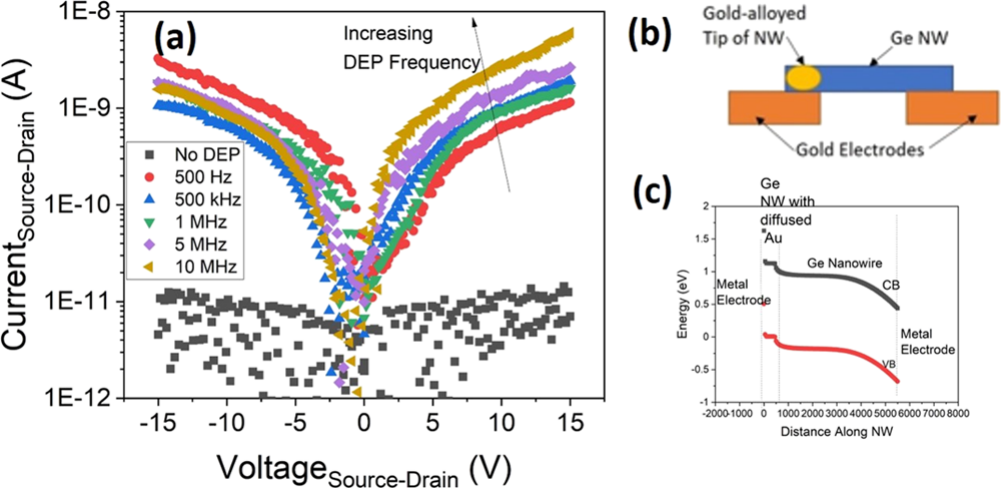
(a) IV characteristics of as-fabricated Ge nanowire FETs at differing DEP frequencies with an ungrounded gate potential, (b) representation of contacted NWs, and (c) schematic of the resulting band alignment.

To determine the optimum DEP frequency, devices were fabricated utilizing a sinusoidal signal with a peak-to-peak voltage of 8 V at frequencies of 500 KHz, 1 MHz, 5 MHz, and 10 MHz. See the Supporting Information for reference measurements to ensure DEP was occurring. Once fabricated, the resultant current–voltage (IV) response was measured for each device at room temperature with no potential applied to the gate electrode (gate left ungrounded). It was chosen to leave the gate with no applied potential as in the final sensor application; the gate voltage will be provided by the bound charge on the surface of the NWs rather than a conventional gate-to-source voltage. As an initial test of the devices, plots of the drain current as a function of the gate-source voltage are provided in the Supporting Information. For each frequency, at least six devices were fabricated. Figure 2 shows the typical IV response (*I*_ds_ vs *V*_ds_) for each DEP frequency. The current is plotted as the absolute current to enable comparison on a log-linear scale; for the measurements, the voltage was first scanned from zero to +15 V and then from zero to −15 V.

For the device without any DEP being applied, there is a slight, negligible increase in the current as the potential is increased. This is most likely due to residual surface contaminants residing in the channel from drop-casting the NWs forming a weak conduction channel. Alternative possibilities may be a chain of overlapping NWs, or a weak conduction path through SiO_2_ to the underlying substrate. However, the current without any DEP signal being applied is at least an order of magnitude smaller than that observed once a DEP voltage is applied.

When a DEP signal is applied, at 500 kHz, a clear IV response can be observed. The response shows a slight asymmetry, and no saturation in the current is observed up to the maximum voltage applied in either direction.

For a fixed bias, in the forward direction, the current increases with the frequency, while the opposite occurs at reverse bias. In both voltage directions, the increase/decrease in current is not a fixed shift with frequency, with a degree of overlapping in the IV response from the devices fabricated at 500 kHz, 1 Mz, and 5 MHz. The reason for this could be the slight differences in the random distribution of NWs used in each devices. However, looking across the entire frequency range, there is a clear change in the asymmetry at the highest frequencies.

As the frequency is increased, influence from electro-fluidic effects in solution reduces and DEP becomes the dominant force in the system, enabling more NWs to be aligned.^24^ This would be expected to manifest as an increase in current across all biases. However, while we see this for positive bias, we see the opposite effect for negative bias potentials. This indicates that the behavior observed here is more complex. It is known that above a certain critical frequency, the DEP force reduces; at this point, only the most conductive NWs will be able to respond to the field.^10^ While intuitively this may be expected to manifest as a reduction in the current at higher frequencies, with the fact that this subset of NWs is more conductive (and has less defects), a further increase in the current may still be observed.

No saturation of the current is observed for all DEP frequencies as the voltage is increased in both directions, as would be expected for a transistor-type behavior. Furthermore, as mentioned, there is a degree of asymmetry in the IV responses. While the device structure is nominally symmetric, the Au nanoparticles used as seeds for NW growth remain on the tip of the NW itself. The observations with TEM show that Au diffuses inside the NW,^25^ resulting in NWs having an alloyed Ge–Au contact at one end, while the other end has an abrupt junction between the Ge of the NW and the Au of the electrode. The result is one contact being Ohmic, while the other is a Schottky contact, as shown schematically in Figure 2. The resultant IV curves in Figure 2 seem to indicate that there is a preferential alignment of the NWs during the DEP process and that as the frequency is increased, the proportion of NWs aligning in the same direction increases, giving rise to asymmetric devices.

**Figure 3:**
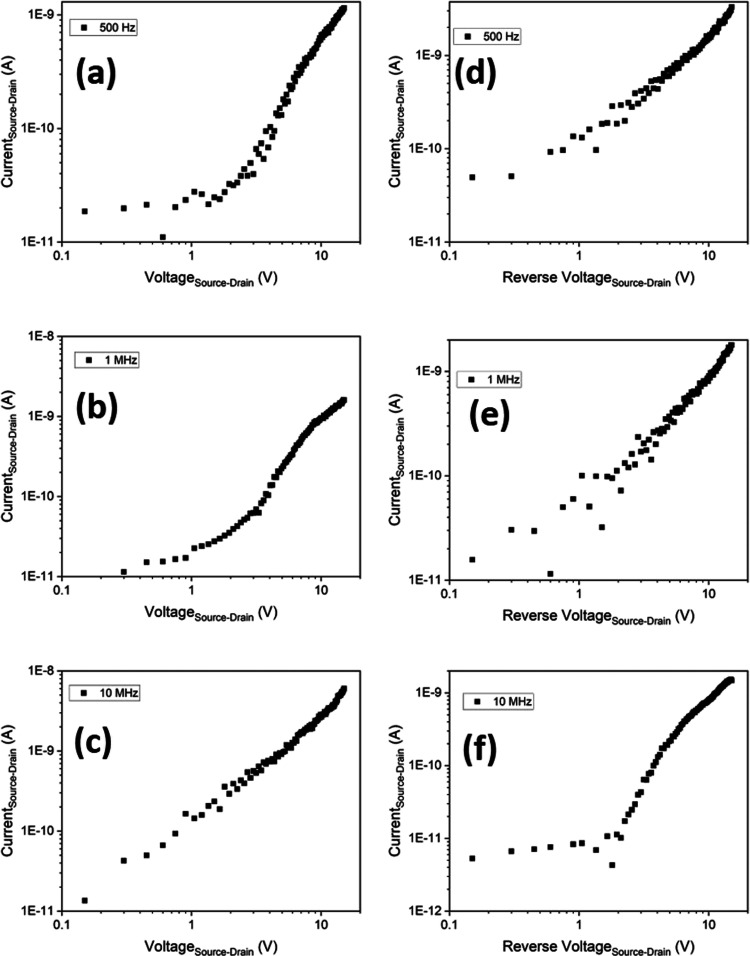
Forward IV characteristics of as-fabricated Ge NW devices fabricated at DEP frequencies of (a) 500 Hz, (b) 1 MHz, and (c) 10 MHz and reverse IV characteristics for devices fabricated at DEP frequencies of (d) 500 Hz, (e) 1 MHz, and (f) 10 MHz, all with a peak-to-peak DEP voltage of 8 V.

To further investigate and understand the performance of devices fabricated at different frequencies, the IV characteristics are replotted on log–log scales for selected frequencies in Figure 3 to enable us to investigate the possible conduction mechanisms in the devices.

Looking first at the device fabricated at the lowest DEP frequency, we observe an initial region (between approximately 0 and 1.5 V) where no current is able to flow, followed by an increase in the current as the voltage is further increased. The gradient of the increasing current is 2.05 ± 0.08, which indicates that the device is dominated by space charge limited currents (SCLC), an effect previously observed in NW-based diodes.^26,27^ While the devices we have here are not strictly diodes, they do have an asymmetric behavior due to a difference in the barrier heights at each end of the NW, and as such, it does not seem unreasonable to extend a similar explanation to this behavior in our devices. At a DEP frequency of 1 MHz, a noticeable change in the IV behavior is observed, with the appearance of three distinct regimes. At voltages up to ∼1 V, there is no significant increase in the current. Between ∼1 and 3 V, the current increases as the voltage is increased with a gradient of 0.85 ± 0.09. This is close to the value of 1, which would be expected for Ohmic conduction. As the voltage is further increased >3 V, the current increases at a faster rate with a gradient of 2.1 ± 0.1, indicating SCLC as the dominant conduction mechanism. For the device fabricated at the highest DEP frequency, significantly different behavior is observed, with the current getting larger as the voltage is increased across the entire source–drain potential range. Only a single gradient is observed for nearly the entire voltage range for this device with a value of 0.95 ± 0.05, indicating Ohmic behavior across the voltage range. The DEP frequencies not shown in Figure 3 for simplicity display the same general trend of IVs becoming Ohmic over a larger voltage range as the DEP frequency is increased.

Conversely, looking at the IV behavior for reverse bias, we observe that for the devices fabricated at 500 Hz and 1 MHz, very similar behavior occurs with an approximately constant increase in the current being measured across the entire voltage range. For these devices, gradients of 1.15 ± 0.03 and 1.20 ± 0.04 were obtained. This suggests that the resultant devices were suffering from high leakage currents. For the 10 MHz device, significantly different behavior is observed; there is initially only a very small voltage dependence on the current that occurs up to a threshold of around 2 V. Above this value, there is a sudden and sharp increase in the current (gradient of 3.75 ± 0.07) to a voltage of approximately 9 V, after which the increase in current begins to reduce. This behavior more closely resembles the one that would be expected from a classical diode in reverse operation with no current flow before a sudden breakdown occurs, although the origin of the breakdown behavior (i.e., Zener, impact ionization, etc.) is not clear. For the devices not shown, a similar behavior is observed to the 500 Hz and 1 MHz case, with only the 10 MHz device exhibiting a significant change to this.

**Figure 4:**
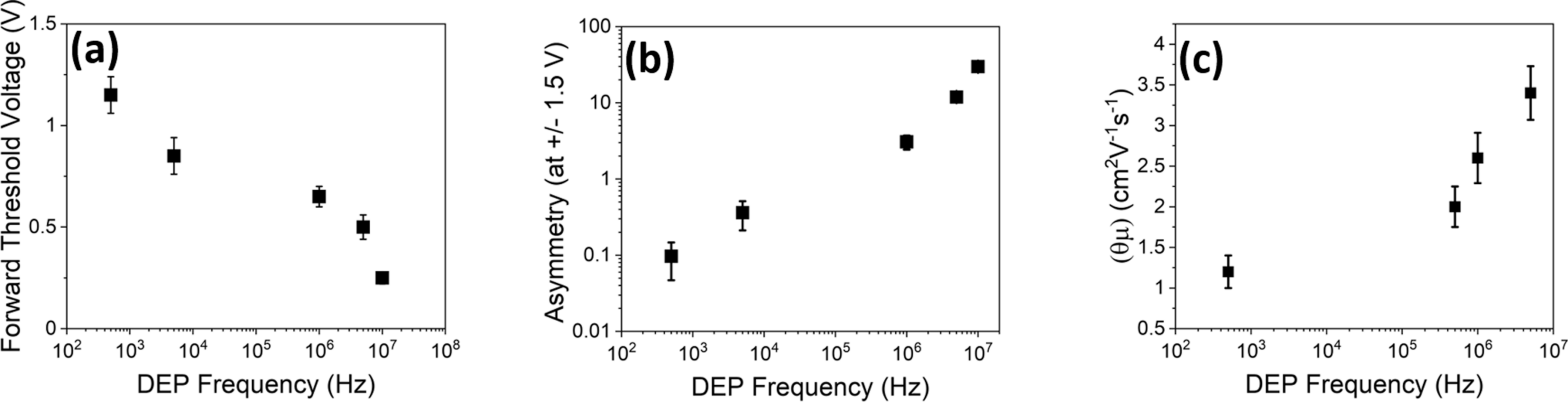
(a) Forward turn-on voltage, (b) asymmetry (at ±1.5 V) as a function of DEP frequency, and (c) (θμ) as extrapolated by fitting the Mott–Gurney law to IV curves as a function of DEP frequency.

To summarize the results and better help in understanding the performance of these devices, Figure 4 shows the “forward threshold voltage” and the asymmetry of the devices as a function of frequency. The threshold voltage was obtained by extrapolating the region of the IVs where the gradient was close to one (or two in the case of the device fabricated at 500 Hz due to the lack of an Ohmic region) back to zero current and using the intercept voltage as the threshold. The asymmetry of the devices is characterized by comparing the ratio of the currents in the forward and reverse regimes at a bias of 1.5 V (this value was chosen, as it is above the turn-on voltage in all devices). For both of these, the values shown in Figure 4 are the average from multiple devices, with the errors given by the standard deviation combined with the error from the initial extrapolation. These show that as the DEP frequency increases, the threshold voltage decreases from a value of 1.3–0.2 V which is close to the expected turn on for a Ge device, while the asymmetry increases by over 2 orders of magnitude over the frequency range.

To understand the cause of the differing device performance, we consider how the DEP force interacts with NWs at each frequency. For the lowest frequencies, the DEP force is relatively weak and likely to be comparable to other electro-fluidic effects in the solution. As such, the NWs may not be fully in contact with the electrodes; resulting in an effective contact resistance. As the DEP frequency is increased, a greater force is applied on the NWs resulting in better adhesion to the electrodes and a reduction in resistance.

Additionally, at low frequencies, a similar force is experienced by all the NWs, regardless of conductivity or number of defects in them. As such, for the lowest frequency devices, all NWs will align and contribute to the observed current. SCLC occurring across all voltages above the turn-on indicates that a significant percentage of the NWs have active traps. As the DEP frequency is increased, the NWs require a higher conductivity to respond to the field; as such, the most defective NWs are removed during the fabrication, resulting in the appearance of an Ohmic region in the IV characteristic between the turn-on and SCLC region.

To further investigate the quality of the NWs for devices fabricated at each frequency, we have analyzed the resultant IV curves using the Mott–Gurney law. If a bulk semiconductor does not have any traps, the SCLC is governed by the V^2^ relationship first derived by Mott.^28^ For NWs which contain charge traps, the law is rewritten in the following form^26,29,30^
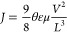
1

In our case, ε is the permittivity of Ge, μ is the carrier mobility on the NWs, *V* is the applied voltage, *L* is the distance between the electrodes, and θ is a scaling constant that is inversely proportional to the density of traps in the NWs. As such, we have fitted the SCLC regions of the IV curves with eq 1 and extracted the product (θμ) as a function of DEP frequency with the results shown in Figure 4c. From the analysis, as the frequency is increased, the product (θμ) also increases. While it is not possible to decouple the terms using this approach, for the product to increase, either the mobility should increase or the trap concentration should decrease or both should occur. An increase in the carrier mobility can improve the quality of the NWs in the device; similarly, a decrease in the trap concentration would signify higher quality NWs. As such, this proves the hypothesis that increasing the DEP frequency results in better quality NWs being selected and aligned.

**Figure 5:**
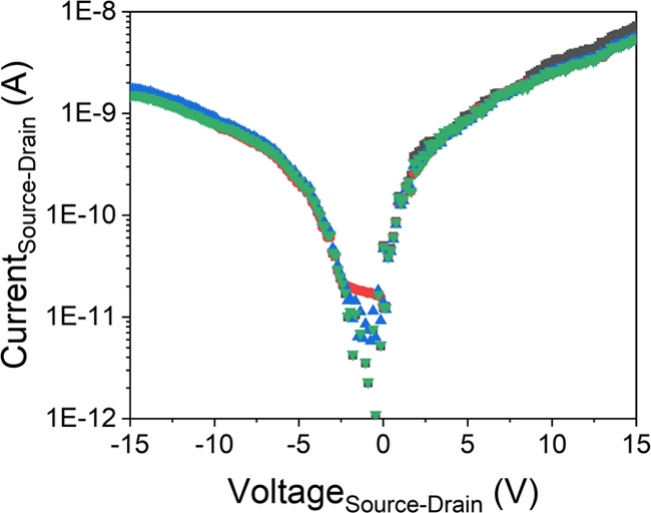
IV characteristics of multiple Ge nanowire devices fabricated with a DEP frequency of 10 MHz and a peak-to-peak DEP voltage of 4 V.

To further investigate the improved performance of devices fabricated at higher DEP frequencies and the potential reproducibility of devices, four repeat devices were made at the highest frequency of 10 MHz. Based on the analysis mentioned above, we expect to only align the most conductive NWs and obtain the most diode-like behavior. The resultant IVs are shown in Figure 5, each separately fabricated spanning a period of several months. Three of the devices show almost identical IV characteristics, with the fourth only showing a slight difference, primarily in the reverse voltage direction. All devices exhibit the same slight asymmetry, as observed previously in Figure 2, and analysis of the forward bias response shows the same behavior as observed previously, with identical gradients, turn-on voltages, and rectification ratios (within the fitting uncertainty) being obtained for all devices. This confirms that despite the different oxide thickness observed in NWs of different ages, the Ge NWs are stable and suitable for device fabrication months after their growth. Furthermore, it demonstrates that DEP is a viable and attractive route to fabricate predictable devices utilizing semiconductor NWs. The batches of NWs used to fabricate each device would have had inherent differences due to age, variation in density, and distribution; however, DEP has enabled the same subset of NWs to be selected each time, resulting in a similar number of NWs with the same mobility in the end device.

**Figure 6:**
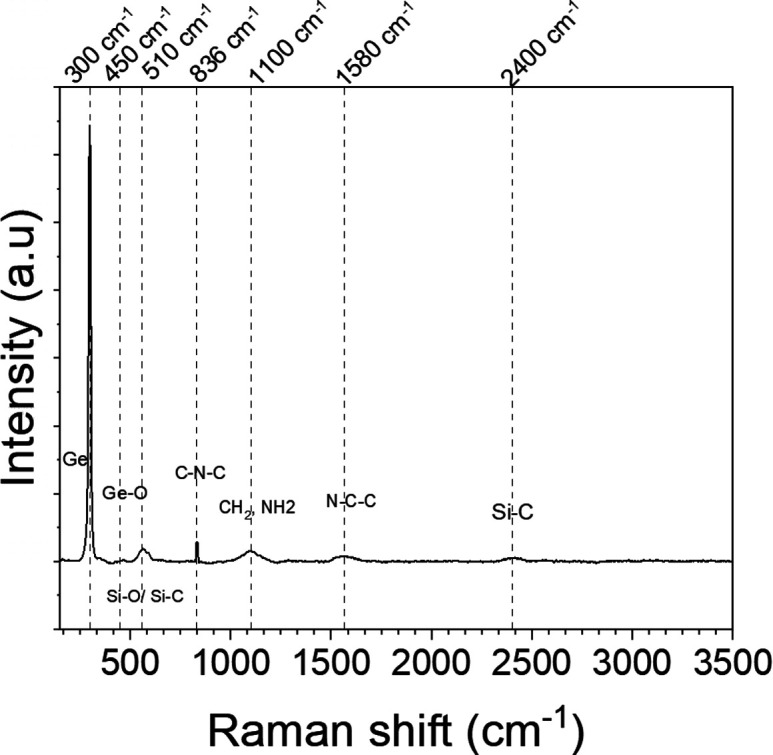
Raman spectra for the Ge nanowire device after a complete functionalization process.

For evaluation as a potential biosensor platform, a device was fabricated using a DEP frequency of 10 MHz and a peak-to-peak voltage of 8 V. The device was functionalized, before being exposed to increasing concentrations of spike protein suspended in PBS. To verify that functionalization was successful and that the aptamers had attached to the NWs, we performed Raman spectroscopy after each step of the process. Figure 6 shows the final scan after the aptamers were attached.

The vibrational band observed at 300 cm^–1^ is attributed to crystalline Ge and at 450 cm^–1^ corresponds to Ge–O from the silane bond. The band observed at 510 cm^–1^ can be attributed to the APTES and originates from the stretching mode of Si–O and Si–C bonds. The presence of the aptamers can be identified due to the stretching mode C–N–C of the amino acid observed at 836 cm^–1^ (strong vibrational signal) and the weak band observed at 1580 cm^–1^ corresponding to the N–H amide bending that can also be associated with the aromatic ring of amino acids. Hence, this demonstrates that the functionalization layers have been successfully attached to the NWs.

**Figure 7:**
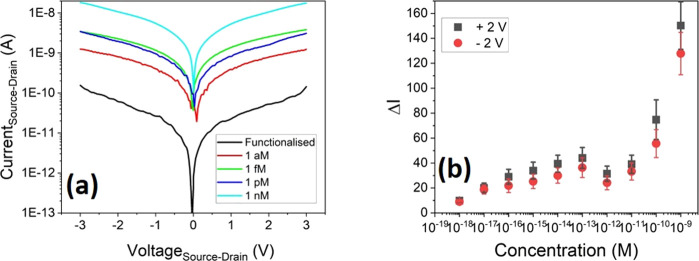
(a) IV characteristics of the as-fabricated Ge nanowire FETs; after functionalization and exposure to increasing concentrations of spike protein, all devices were fabricated with a DEP frequency of 10 MHz and (b) relative increase in the source–drain current, relative to a functionalized device at a bias ±2 V.

Four devices were then fabricated using the same procedure, with IV curves being measured after fabrication and upon exposure to increasing concentration of spike protein. This was achieved by drop-casting a small volume of spike protein dispersed in buffer solution onto the device and allowing it to dry to perform in-air measurements (see Supporting Information for full details). Figure 7a shows the resultant IV curves (absolute value of the source–drain current as a function of the applied source–drain voltage) of one device for each concentration, along with a functionalized device without protein present. After the device is functionalized, there is a significant reduction in the current at all biases compared to the unfunctionalized device. The presence of the bound aptamers on the NWs has introduced a charge distribution across the NW surface, which in turn results in an induced charge depletion within the NWs and the observed current reduction. This behavior has previously been modeled by adding p-type charges to the surface of NWs^31^ and as such suggests that the overall net charge from the aptamers used here is p-type.

An increase in the measured current is observed at all biases upon exposure to the spike protein. This is due to the attachment of the spike protein to the aptamer, resulting in a change in the charge distribution at the surface of the NWs, creating an effective applied gate charge. To ensure the changes in the IV response are due to the protein binding and are repeatable, two reference devices were made and tested (see Supporting Information). Previous simulations on Ge NWs have shown that, if molecular charge transfer of holes with a density of 10^18^ cm^–3^ occurs from the surface of the NW, this could result in a theoretical enhancement of up to 400% in the current under a bias of 0.1 V.^31^ The results we observe here suggest that a similar transfer of charge may occur from the protein to the NWs. To quantify the changes in current with the concentration of the spike protein, the relative change in current at a fixed bias point (±2 V) is plotted as a function of the concentration, as shown in Figure 7b. The parameter Δ*I* is used to analyze relative change in the current given in eq 2 below.
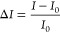
2where *I* is the measured current and *I*_0_ is that measured at the same voltage without the presence of the target protein. While we have used a bias of 2 V here, a similar effect is seen for all bias points (positive and negative) measured.

Between concentrations of 1aM and 100 fM, we see a clear increase in the measured current as the spike protein concentration is increased, with a gradient of (5.1 ± 0.9 for the +2 V points and 6.3 ± 0.8 for the −2 V points). This indicates that over this range, the amount of protein binding to the aptamers is increasing, resulting in a changed surface potential and hence a change in the measured current. Between a concentration of 100 fM and 10 pM, a saturation region is observed; this seems to indicate that once a concentration of 100 fM has been reached, all the aptamers bound to the NWs have now bound to a protein. For concentrations above 10 pM, a very sharp increase in the current is observed. The origin of this mechanism is not clear. However, it may be that at such high concentrations, current is able to travel directly through the protein layer itself; an alternative explanation may be that as the entire substrate is functionalized (SiO_2_ and NWs), a conduction path is formed around the NWs by the protein/aptamer layers, or that charge transfer through proteins allows NWs to transfer charge between themselves.

**Figure 8:**
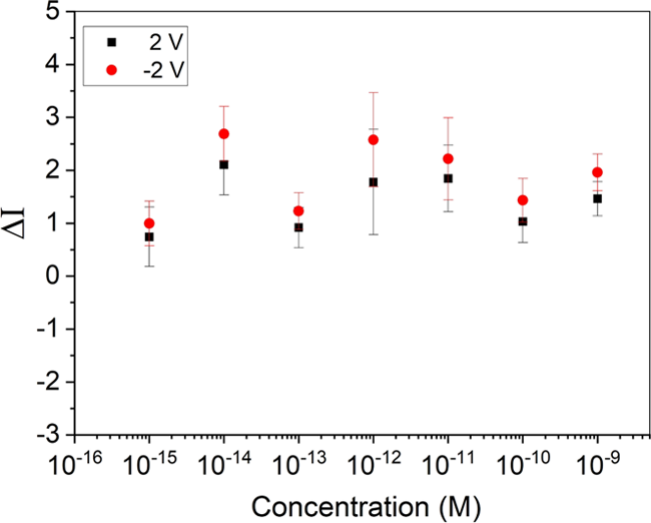
Relative increase in the source–drain current, relative to a functionalized device at a bias of ±2 V for exposure to increasing concentrations of BSA.

To ensure that the devices have an appropriate selectivity to the spike protein, tests were conducted using bovine serum albumin (BSA) on the four devices, using the same method as before, and the same ratio as defined in eq 2 was calculated. Figure 8 shows the resultant ratio for increasing BSA concentration. No discernible response is observed across the range, demonstrating that the functionalized NWs have a good selectivity to spike protein.

There have been several other reports recently looking at FET-based biosensors for the detection and monitoring of COVID-19.^32−34^ These have all shown similar responses in terms of sensitivity to spike protein concentration. Our previous work^33^ reported results similar to those shown here in terms of using aptamers rather than antibodies and by performing the measurements in air rather than in liquid. The key difference in this work is in the use of Ge NWs as the active channel rather than bulk silicon. Compared to our previous result, the use of NWs has resulted in enhanced sensitivity, with increased current being observed at lower concentration here. This improvement is due to the reduced size of the NWs compared to our previous channel width in a Si-based FET, with a bound protein constituting a larger percentage of the NW surface compared to the previous channel. As mentioned previously, this suggests that a device consisting of just a single NW may provide the ultimate limit of detection for such a sensor system.

Similarly, there have been previous studies looking at utilizing NWs for biosensor applications, for a range of potential targets. As discussed earlier, NW-based bio-FETs that have been reported to date have used either drop-casting of dispersed NWs between contacts or expensive nano-fabrication to realize devices. Here, we have demonstrated that DEP is a viable alternative approach to fabricated NW bio-FETs, yielding reproducible and reliable device performance.

In summary, we have demonstrated that DEP can be used to reliably and repeatedly fabricate NW-based devices with the same electrical characteristics each time, demonstrating this as a viable manufacturing approach. Using a combination of Ge NWs and a known aptamer sequence that binds to the spike protein of SARS-Cov2, we have demonstrated a NW aptamer sensor capable of performing in-air measurements. This has demonstrated a linear response over the concentration range from 100 fmol/L to 1 nmol/L, indicating a lower detection limit than was previously observed for in-air measurements of COVID-19 spike protein.

## Methods and Experimental Details

### Ge NW Growth

In the following work, Ge NWs with lengths exceeding 20 μm are grown via MOVPE on Ge substrates utilizing gold (Au) nanoparticles as seeds. Samples were grown over a period of 1 h in a standard hot wall metal–organic vapor-phase epitaxy (MOVPE) at a pressure of 100 mbar and using^25^ iBuGe as a precursor, kept at a constant temperature of 5 °C within a thermostatic bath.^35^ Within the chamber, palladium-purified H_2_ was used as a gas carrier. The NWs have been characterized by SEM in order to check their quality and dimensions. As discussed in ref (25), the length of these NWs can be controlled by changing the growth time, while maintaining a highly reduced degree of tapering (increase of NW diameter over the length). Prior to analysis, they were transferred to a carbon-coated Cu grid by applying gentle scrubbing. The NW growth axis is along the ⟨111⟩ direction, and they are measured to be 20–25 μm long with an average diameter of (60 ± 5) nm, as measured by SEM. The amorphous oxide layer at the NW surface has a thickness ranging between 0.5 nm (fresh NWs observed just after growth) and 2.0 nm (aged NWs observed after 2 months in air). Further, the NW tip composition has been analyzed by energy-dispersive X-ray spectroscopy (not shown here), and it was found that the tip contains Ge and Au with a composition of (27 ± 3) atom % Ge and (73 ± 3) atom % Au, as estimated from Ge-L and Au-M lines, close to the eutectic composition.

### Device Fabrication Via DEP

The full fabrication procedure is provided in the Supporting Information. In brief, the Ge NWs were removed from the substrate via sonication and then drop-cast between two electrodes while applying a sinusoidal voltage. This gap size was selected, as it has been shown that NWs with a length similar to the electrode gap undergo an optimal DEP force interaction.^36^

### Functionalization

After the DEP process, the aligned NWs were functionalized in order to attach the binding element to their surface. The full procedure is given in the Supporting Information. In brief, the aptamer probe molecule was attached to the germanium surface using a silanization method.^37^ The functionalized devices were subsequently immersed in a solution containing amine-terminated aptamers (Eurogentec, Belgium). An aptamer previously reported to bind to the spike protein of the SARS-CoV2 virus (MERCK) was used.^38^

They were subsequently rinsed in PBS (MERCK) and dried in a N_2_ atmosphere to remove excess material as well as any water in the layers. Figure 1c provides a schematic overview of the final functionalized device.

To investigate the devices’ response to the protein, increasing concentrations (100 fM to 1 nM) of spike protein (Cambridge BioScience) dispersed in PBS were drop-cast onto the sample and the current–voltage response was measured after each exposure.

## Supporting Information Available

In situ impedance monitoring for the electrode gap during the DEP process, showing NW alignment occurrence, transfer characteristics of fabricated un-functionalized devices, and control tests on a device with no NWs between the electrodes and showing the effect of multiple rinses in PBS on a fabricated device (PDF)
