# Supplementary material for: Selection and Functionalization of Germanium Nanowires for Bio-Sensing
Source: ACS Omega. 2022 Sep 23;7(39):35288–96. doi: 10.1021/acsomega.2c04775 (PMC9535719; doi:10.1021/acsomega.2c04775)
Supplement: Supplementary file 1 — ao2c04775_si_001.pdf [file ao2c04775_si_001.pdf]

Supplementary Information

Selection and functionalization of germanium nanowires for bio-sensing

Siriny Laumier[1], Thomas Farrow [1], Harm van Zalinge[1], Luca Seravalli[2], Matteo Bosi [2] and Ian Sandall [1,*]

[1] University of Liverpool, Department of Electrical Engineering and Electronics 9 Brownlow Hill, Liverpool L69 3GJ, UK
[2] Institute of Material for Electronic and Magnetism Parco Area delle Scienze 37/A 43124 Parma, Italia

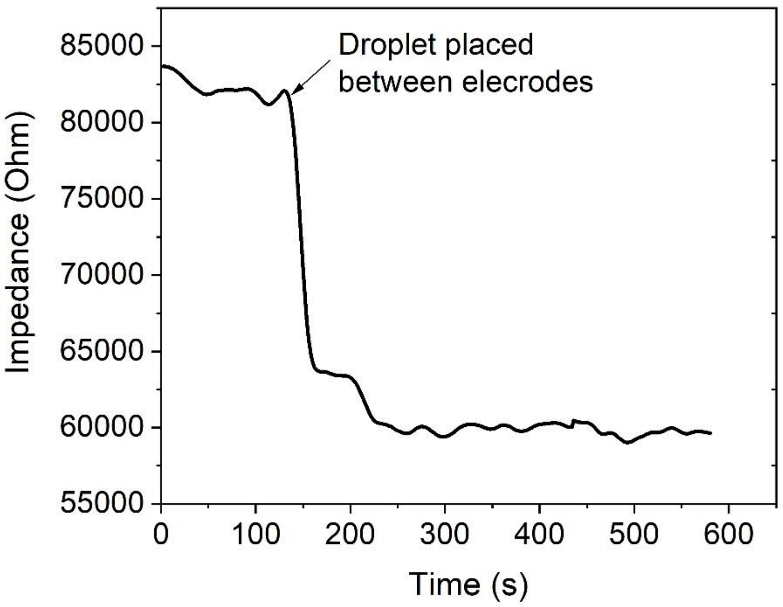

Figure S1 – Example plot of in-situ monitoring of resistance as a function of time during the DEP process. Taken from a device fabricated with a DEP frequency of 10 MHz. Arrow indicates the time when the NWs dispersed in solvent were drop cast between the electrodes.

As the NWs align, they form an electric bridge between the contacts and hence cause a drop in the observed impedance, verifying that alignment has occurred.

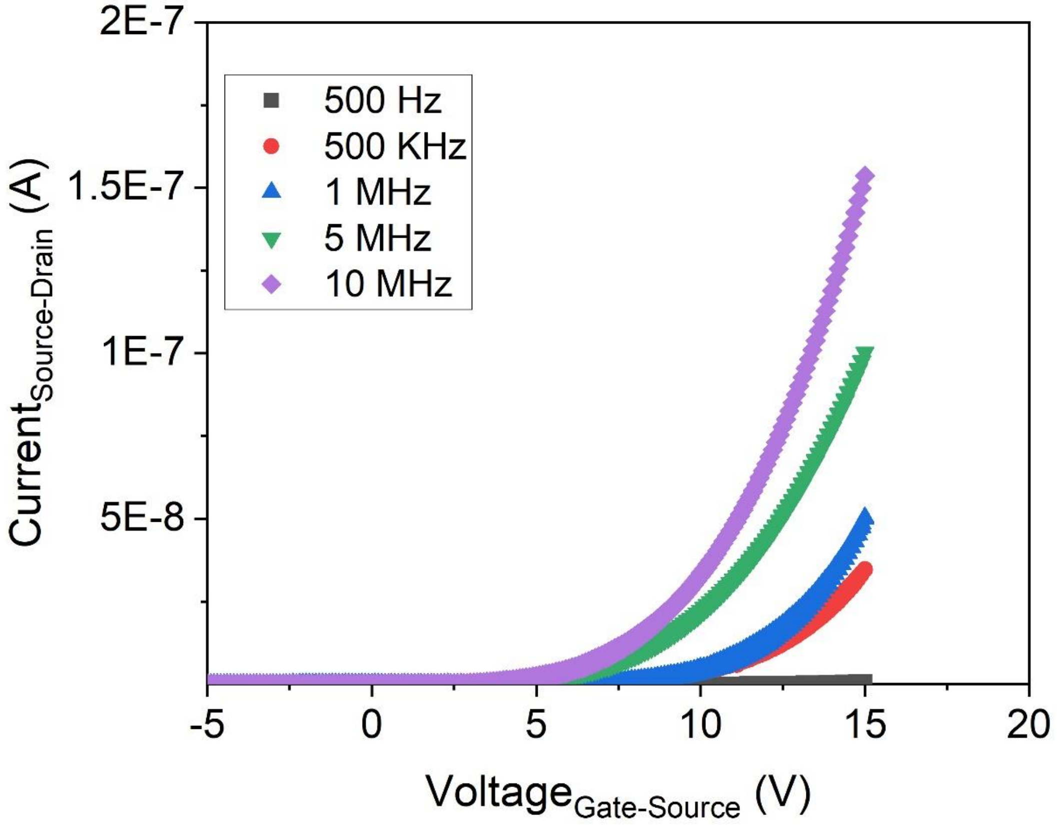

Figure S2 – Transfer Characteristics (V_{gs} Vs I_{ds}) for unfunctionalised nanowire devices fabricated at different DEP frequencies. All curves are shown for a fixed drain-source voltage (V_{ds}) of 2 V.

Figure S2 shows the transfer characteristics (V_{gs} vs I_{ds}) at a fixed drain source voltage (V_{ds}) of 2 V for unfucntioanlised devices fabricated at increasing DEP frequency. A clear trend is observed with an increasing current as the frequency is increased, indicating a higher DEP frequency results with more classical device performance.

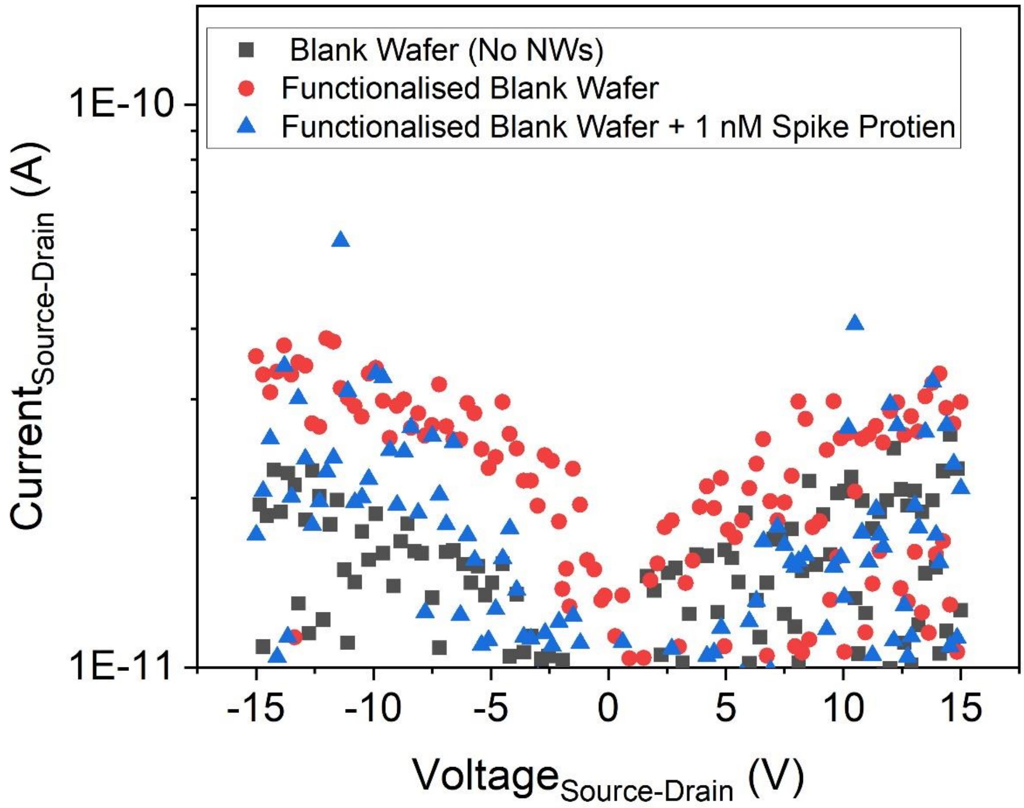

Figure S3 – Control test showing current-voltage curves from a blank substrate (with no NWs), pre and post functionalisation as well as after functionalisation and exposure to 1 nM of spike protein. There a slight increase in the current as a function of bias as discussed in the paper after functionalisation there is potentially a further increase in current by about a factor of 2 but this is still over an order of magnitude below the current in NW devices reported in the paper.

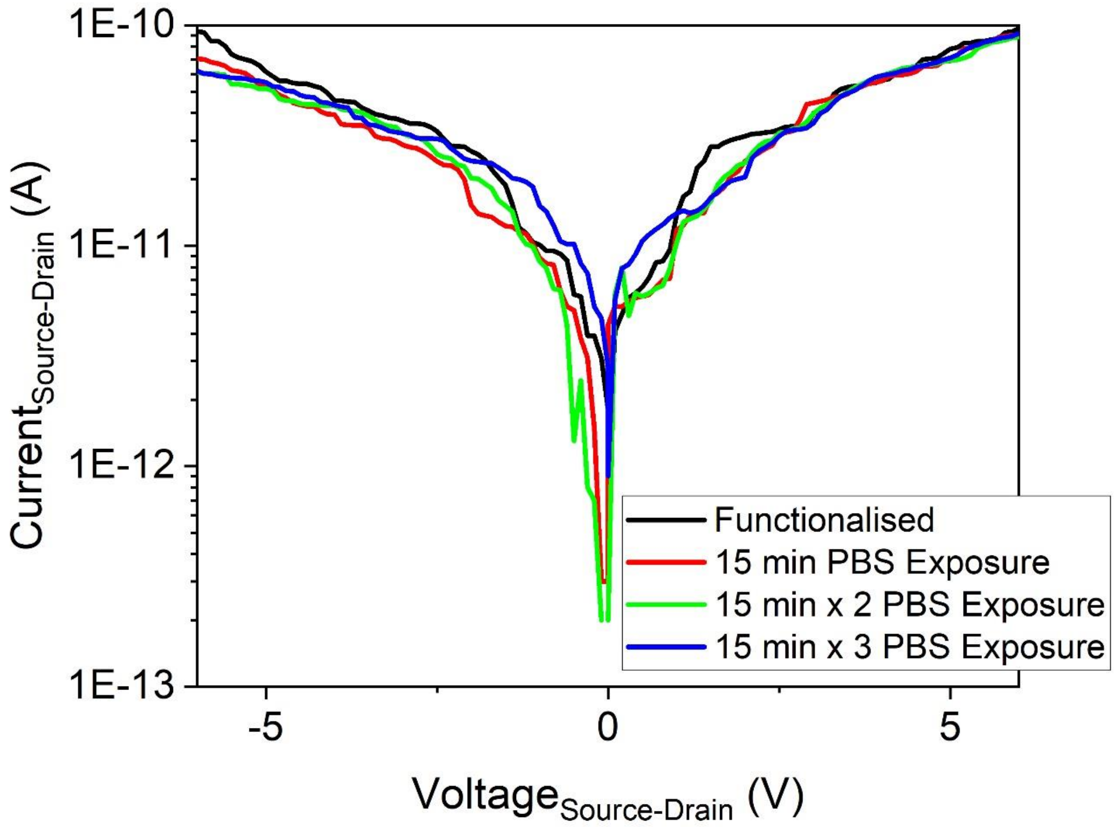

Figure S4 – Control test showing current-voltage curves from a functionalised device fabricated with a DEP frequency of 10 MHz, after multiple exposures to PBS solution (with no spike protein), sample was rinsed and dried between each exposure. The results indicate that there is no change in the device performance due to PBS exposure and as such results reported in the paper are due to the presence of the spike protein.

Figure S3 shows a reference devices with no NWs (i.e. bare electrodes) and is used to verify that the current measured is due to the NWs and not charge transport through aptamers / protein layers. This device exhibited no measurable current at any bias or protein concentration. Figure S4 shows the results from a second reference device indicating that the functionalised nanowires were stable. The device was exposed to PBS for increasing lengths of time and the current-voltage response was collected using a probe station system at room temperature in air, with no change in the electrical performance being detected. From the result of these two reference checks we are confident that the current measured in this work is due to conduction through the NWs and the changes with spike protein concentration are caused by the variation in the surface potential.

Full Device Fabrication Procedure Using DEP

The Ge NWs were removed from their substrate by low power sonication (~100 W) for a duration of 60 s and suspended in anisole to enable DEP process. Source and drain contacts (also used as electrodes for the DEP) were defined and deposited onto insulating substrates wafer (500 µm SiO_2 on P doped Si, provided by University Wafer) via standard microfabrication techniques. All chemicals and metals used in this work were obtained from Merck and used as-received unless otherwise stated. A layer of 5 nm of chromium followed by 50 nm of Au was deposited by metal evaporation, photolithography was used to define the electrode geometry, undeveloped resist was then removed before the samples were placed in Au etchant to remove the unwanted metal. The resist was subsequently removed by immersing the substrate into acetone. The electrodes were defined to have a central gap of 20 µm to coincide with the mean nanowire length, as determined from SEM analysis. This gap size was selected as it has been shown that NWs with a similar length size to the electrode gap undergo optimal DEP force interaction [33]. The tips of the electrodes were defined to be rounded to prevent any abrupt changes in the electric field profile due to corners during the DEP process. Figure 1, shows a schematic of the DEP system, along with the final device configuration.

Prior to the NWs solution being drop-cast between the electrodes, a sinusoidal voltage was applied, for the DEP process. A droplet of 2 µL of NWs solution was placed between the electrodes, with the sample held at a small angle (~10°) to enable a roll flow of solvent and NWs towards the electrode gap. The alignment process was carried out for ten minutes until the solvent was fully evaporated. Afterwards, the electrodes were gently rinsed with isopropanol and blow dried with nitrogen to wash out impurities and any non-attached NWs (i.e. NWs shorter than 20 µm or with low conductivity so not fully aligned to the electrodes).

Nanowire Functionalization Procedure

Once the optimum DEP parameters had been determined, a series of fresh devices were fabricated to investigate their functioning as potential biosensors. After the DEP process, the aligned NWs were functionalized in order to attach the binding element to their surface. The aptamer probe molecule was attached to the germanium surface using a silanization method [34]. In brief, the wafer was immersed in an ethanol solution containing 3% (v/v) (3-aminopropyl) triethoxysilane (APTES) at 80 °C. After 2 hours the sample was removed from

the solution and washed four times with ethanol to remove any excess material. This step was completed by drying the sample under nitrogen and a curing step of 1 hour at 110 °C. The surface at this point is terminated with an amine group that can be used for further functionalisation.

The samples were then immersed in an aqueous solution containing 2% (v/v) glutaraldehyde at room temperature ((25 ± 2) °C) for 1 hour. The glutaraldehyde acts as a crosslinker, binding with the amine terminated silane and providing an aldehyde binding group for the amine terminated aptamer sequence. After rinsing, the sample was dried under N_2. The functionalized devices were subsequently immersed in a solution containing amine-terminated aptamers, Eurogentec, Belgium. The device and solution (200 µL, 100 nM), were incubated at 37 °C for 2 hours and then rinsed with 1× phosphate-buffered saline (PBS) and deionized water to remove the excess aptamers and subsequently dried under N_2. An aptamer previously reported to bind to the spike protein of the SARS-CoV2 virus was used [35]. The specific sequence is 5'-CAGCACCGACCTTGTGCTTTGGGAGTGCTGGTCCAAGGGCGTTAATGGACA-3' with an amine group attached to the 5' end.

There is a possibility that not all aldehyde groups provided by the glutaraldehyde will have bound to an aptamer and as such, they would provide sites that potentially can bind non-specifically to any amine group present in the proteins. To prevent this, following the aptamer functionalization, the samples were immersed in a PBS solution containing 80 mM glycine for 60 minutes at room temperature. They were subsequently rinsed in PBS and dried in a N_2 atmosphere to remove excess material as well as any water in the layers, figure 1c provides a schematic overview of the final functionalized device.

To investigate the devices response to the protein, increasing concentrations (100 fM to 1 nM) of spike protein, Cambridge BioScience, dispersed in PBS were drop-cast onto the sample and the current-voltage response was measured after each exposure. For all concentrations, a droplet of 4 µl was deposited on the functionalized NWs and left for 15 minutes, the sample was then washed using PBS to remove any unbound protein and gently blown dry with nitrogen, prior to measurement.
